# Cloning of cellulase gene using metagenomic approach of soils collected from Wadi El Natrun, an extremophilic desert valley in Egypt

**DOI:** 10.1186/s43141-022-00312-9

**Published:** 2022-02-08

**Authors:** Safaa M. Ali, Nadia A. Soliman, Samia Abd Allah Abdal-Aziz, Yasser R. Abdel-Fattah

**Affiliations:** 1 Nucleic Acid Research Department, Genetic Engineering and Biotechnology Research Institute (GEBRI), City of Scientific Research and Technological applications, Alexandria, Egypt; 2 Present address: City of Scientific Research and Technological Applications (SRTA-City),, New Burg El-Arab City, Universities and Research Institutes Zone, Alexandria, Post 21934 Egypt; 3 Bioprocess Development Department, Genetic Engineering and Biotechnology Research Institute (GEBRI), City of Scientific Research and Technological Applications (SRTA-City), Alexandria, Egypt

**Keywords:** Cellulase, Cloning, Degradation, Expression, Extremophilic, Metagenome

## Abstract

**Background:**

Wadi El Natrun microorganisms have been considered as a new resource for natural products due to its extreme condition of salinity and alkalinity. Therefore, this study was devoted to generate metagemic library from soils collected from such an extreme environment in order to clone a novel cellulase for physique industrial applications.

**Results:**

Total soil-DNA was successfully extracted, and then digested by different restriction enzymes. Purified fragments ranged ~ 200–6500 bp were ligated and were cloned into plasmid cloning vector (pUC19) by using *Escherichia coli* DH5α (*E*. *coli*) host cells. A constructed metagenomic library composed of 270 clones was screened on carboxymethylcellulose (CMC) agar plate where the active clones had been characterized by the formation of the yellowish halo zone. Thereafter, clone 1 was selected as the most active as being based on cellulase activity quantification (19 μ/ml). Plasmid related to clone 1 encoded *cellSNSY* gene of approximately 1.5 kb was subjected to molecular characterization; the obtained partial sequence of 861 bps encoded 287 amino acids showing 76% similarity to the endoglucanase gene of *Bacillus amyloliquefaciens*. The recombinant *cellSNSY* was expressed under *lacz* promoter at 1 mM of isopropyl β-d-1-thiogalactopyranoside (IPTG), giving 21 μ/ml cellulase after ~ 27 h. Sodium dodecyl sulfate–polyacrylamide gel electrophoresis (SDS-PAGE) and an activity staining of the recombinant *cellSNSY* which revealed an active band with a molecular mass ~ 59 kDa appeared in the induced sample. The maximum enzyme activity of crude *cellSNSY* was observed at 45 °C and for a pH of 8.5. Interestingly, the enzyme activity was slightly inhibited by ethylenediamine tetraacetic acid (EDTA) and methanol. It showed high resistance to the tested heavy metals and the surfactant which ordered Zn> (SDS,Fe)>Mn>Cu.

**Conclusions:**

This study established an easy and a skillful way to clone/express a new found cellulase gene(s) under *lac*Z promoter. The isolated recombinant *cellSNSY* showed 76% similarity to endoglucanase gene, and the enzyme showed tolerance to the mostly tested agents including heavy metals, surfactant, solvents, and EDTA. Additionally, the studied recombinant showed a high stability up to 55 °C and for alkaline pH 8.5. These features make it an ample and viable for many applications.

## Background

Cellulose is the most cost-effective, natural, and renewable organic compound used as a carbon source in an assortment of solicitations [1]. The much more common carbohydrate on the planet is cellulose. It is the primary component of plant materials. It can be found in a multiplicity of dwellings including wood and plant cell walls, bacteria, algae, and tunicates which are the only ones that have it. This abundant supply naturally enables the development of novel customs for this adaptable material [2]. It is an extensive chain of glucose units linked by 1,4 glucosidic connections [3, 4]. Cellulosic materials have been used for a number of devotions in recent decades including biomedical applications [5, 6].

Chemical and enzymatic hydrolysis are the two methods for cellulose to glucose conversion (Cellulases are used as an enzymatic method for this resolution and are known as an eco-friendly process because it does not produce secondary polluting metabolites) [7–9]. Cellulases, in specific, are accountable for cellulose decomposition by hydrolyzing the 1,4-glycosidic linkages [10]. Cellulases are one of the greatest recurrently used industrial enzymes with over 30 years of commercial handiness [11]. These are inducible enzymes bent by a variety of microorganisms, such as bacteria and fungus, when growing on cellulose materials. Cellulase is a multifunctional enzyme that combines exoglucanase, endoglucanase, and d-glucosidase to make a public announcement little expanse of glucose. As a result, cellulose is converted to glucose, a simple sugar that can be fermented into cellulosic biofuels [12]. Animal and plant enzymes are less stable than microbial enzymes. They have a number of advantages including the aptitude to be manufactured at a lower cost and in a shorter time frame using fermentation processes with high consistency, as well as the ability to readily optimize the process [13]. As a result, cellulases enzymes are an option in a wide range of industrial situations including the paper and the pulp industries, textile and bioethanol industries, wine and brewery industry, food industries, animal feed industry, agricultural and detergent industries [11, 14–16], pharmaceutical industries, and the handling of waste [17, 18].

In biotechnology, extremophilic bacteria have a wide range of current and potential applications. The diversity of prokaryotic populations was investigated in the water and from the sediments of three largest lakes in the Wadi El Natrun [19].

The scarcity of novel cellulases with diverse features has evolved into a bottleneck in the effectual cellulase use. The term “metagenome” refers to a group of genes discovered in the environment with foreseeable vast repositories of cellulases. New cellulases, on the contrary, are difficult to discover by using functional metagenomic library screening. Since the technique is based on the cellulase activity rather than on the sequence similarity. Consequently, finding new cellulases via functional screening of metagenomic libraries is difficult in nature. The targeted selection of novel cellulase sequences for high-throughput expression is possible with metagenomic sequencing. Next-generation sequencing, such as metagenomics, has revolutionized the study of the “unseen majority.” Metagenomics application accustomed to investigate new enzymes is important to result for permitting researchers to get the knowledge about the diversity of the microorganisms with reaching for 99% and for numerous sorts of genes coding catalyst that have not nonetheless been known [20, 21]. Metagenomics has been thought-about as an economical approach for locating novel cellulase/hemicellulase from microbes, particularly ingenuous microbes [22]. This method allows us direct access to bioactive candidates as well as in-depth study of microbial genomes. The current metagenomic research of new enzymes and systems that serve as hosts opens a Pandora’s Box for improving the bioenergy industry, which plays a significant part in the bio-economy [23].

The objective of the present study aimed to clone functional gene(s) degrading cellulose using soil-metagenomics from Wadi El Natrun located in Egypt due to its extreme conditions. The process started by soil collection, total deoxyribonucleic acid (DNA) isolation, fragmentation, cloning, and then, expression of the isolated gene(s) in a suitable host cells (*E*. *coli* DH5α). Subsequently, molecular and functional characterization for cloned gene was investigated.

## Methods

### Sample collection and preparation

The soil samples (2) were collected from Alexandria (2018), Egypt’s Wadi El Natrun (Latitude & Longitude (WGS84): 30° 27′ 20′′ North, 30° 10′ 20′′ East), secondly were transferred to the laboratory in ice box, thirdly were placed in a deep freezer (− 20 °C), and then were inspected.

### Chromosomal DNA preparation

DNA isolation from soil sample was carried out using soil-isolation DNA kit (QIAGEN). In brief, the soil sample (500 mg) was suspended in 10 ml of 0.1 M sodium phosphate buffer (pH 8) before being added to 0.5 ml of 20% sodium dodecyl sulfate (SDS) and was mixed numerous times by inversion. Afterwards, 20 μl lysozyme (10 mg/ ml) was added and was mixed well (30 s); then the protocol of DNA isolation was completed according to the manufacturer’s recommendation. Afterwards, the purity and the concentration of the isolated DNA were detected by using NANO DROP (Thermo Scientific™ NanoDrop 2000).

### DNA restriction digestion

The isolated soil- DNA was digested in 20 μl reaction volume; 2 μl of enzyme buffer and 1–2 U restriction enzyme were added. DNA digestions with restriction enzymes were performed under the reaction conditions specific for each enzyme, as being suggested by the manufacturers (Fermentas). Different restriction enzymes were used (*BamHI*, *EcoRI*, *HindIII*, and *SalI*) for the DNA digestion and the cloning vector (pUC19), as well.

### DNA ligation

A ligation of the digested DNA-vector (ratio 4:1) was made into 20 μl reaction volume using T4 DNA ligase (1U) (Fermentas) and 2 μl buffer as the mixture reaction was kept at 16 °C overnight. PUC 19 (plasmid cloning vectors) was used as cloning and expression vector which allowed blue/white screening for the recombinant clones through *Lac*z promoter in the presence of the lactose analogue (IPTG, 1M) and the chromogenic agent 5-bromo-4-chloro-3-indolyl-β-d-galactopyranoside (X-gal, 200 mg/ ml).

### Competent cells preparation and transformation

*Escherichia coli* DH5α was prepared according to Sambrook et al. [24]; 100 ml Luria-Bertani (LB_low salt_) liquid medium of the following composition (g/l): yeast extract, 10; peptone, 5; NaCl, 5; they were inoculated with 100 μl overnight culture of *E*. *coli* DH5α and were kept warm at 37 °C with shaking (170 rpm) till optical density (OD _600_) 0.6–0.7. The flask was ice-chilled and was aliquoted into 50 ml portions. Cells were collected with centrifugation at 4000 rpm and re-suspended in one ml of transformation storage solution (TSS). This solution composed of (w/v %) polyethylene glycol 6000 (PEG); 10, MgCl_2_.6H_2_O; 1 and 5 ml dimethyl sulfoxide (DMSO), complete to 100 ml final volume with LB broth then adjust the pH at 6.5 as described by Chung and Miller [25].

Cell suspension was dispensed into sterile Eppendorf tube (200 μl aliquots), which was frozen immediately at − 80 °C. Frozen aliquots of the competent cells were allowed to thaw on ice. DNA (ligation mixture) was added to the tube, and then incubated for 20 min on ice. The tube was heat shocked at 42 °C for 60 s then 800 μ1of LB medium was added and then incubated at 37 °C for 1 h with shaking. Aliquots (200 μl) were spread on selective LB plates containing ampicillin (amp), X-Gal, and IPTG with a final concentration % 10 mg, 2 mg, and 1 mM, respectively. A master plate for white clones was prepared to test individually clone ability to degrade the cellulose macromolecule.

### Screening soil metagenomic clones for expressing cellulase gene

To differentiate the positive cellulase producing clones among soil metagenomic clones (white clones), qualitative detection (plate assay) was carried out. The tested clones were screened for cellulase activity in Nutrient agar (NA_amp& IPTG_) supplemented with 0.5% of carboxymethylcellulose (CMC). The positive clones characterize by formation of a yellowish halo around the colonies after addition of indicator (0.2% w/v Congo red) followed by washing with 1 M sodium chloride [26].

### Enzyme colorimetric assay

The cellulase activity was determined by monitoring the released reducing sugar (measured as glucose) upon enzymatic hydrolysis of CMC substrate, by applying the dinitrosalicylic acid (DNS) method and a standard curve was generated using crystalline glucose powder [27]. Cell pellets of positive clones were sonicated in 2 ml phosphate buffer pH 7. A 0.5 ml of tested pellet lysate was mixed with 0.5 ml of the CMC-substrate (0.5% w/v) in tris-HCl buffer (50 mM, pH 7.5); the reaction mixture was incubated for 15 min at 50 °C. Afterwards, the reaction was stopped by adding 1 ml of DNS and boiling for 10 min. The released reducing sugar producing color was measured at 540 nm by spectrophotometer (UV, SHIMADZU); the absorbance was measured against control (without active enzyme). Under standard test conditions, enzyme activity (U) was defined as the amount of enzyme that produced 1 μg of reducing sugar equivalent to glucose per minute. All assays measurements were calculated after subtracting from the individual control which composed of mixture of the enzyme and substrate boiled before being used as a control sample.

### Preparation of plasmid-DNA from E. coli recombinant cells harboring insert

Mini-plasmid extraction was carried out via alkali lysis method as defined by Sambrook et al. [24]. Cells (1.5 ml) from overnight culture of the selected clone were collected by centrifugation at 7000 rpm and were sequentially suspended in three solutions (I, II, III) by equal volume; then, using mild shaking after the addition of solution I& II and standing 15 min after solution III. The first (Tris-HCl-pH 7.5 and 100 mM EDTA) was for solubilizing the cell pellet , the second was for lysis (1 M NaOH and 5.3% (w/v) SDS), and the third (60 ml of 5 M K-acetate and 11.5 ml acetic acid w/v%) was for the precipitation of the protein and the high molecular weight DNA. The plasmid containing supernatant was separated after applying centrifugation for 10 min at 13,000 rpm. Finally, the extracted plasmids were precipitated by isopropanol, were washed with 70% ethanol, and then were suspended in 30 μl water. Polymerase chain reaction (PCR) was applied to amplify the cellulase gene harboured-plasmid by the most active clone(s) using M13 flanking primer (M13F: 5′AGGCCCTGCACCTGAAG3′ and M13R: 5′ TCAGCGCCTGGTACC3′ [28]. The PCR was carried out for 30 cycles at 94 °C (denaturation) for 1 min, 55 °C (annealing) for 1 min and 72 °C (extension) for 2 min, followed by 10 min at 72 °C (final extension). After completion, a fraction of the PCR product was examined by using the agarose gel electrophoresis [29], and the remnant mixture was purified by using the QIAquick PCR purification reagent (QIAGEN) according to the manufacturer’s recommendation. Afterwards, PCR product was subjected to automated DNA sequencing using the ABI PRISM model 3730 [30].

### Sequence similarity

Basic Local Alignment Tool (BLAST) (www.ncbi.nlm.gov/blast) is an algorithm and is an applied program for comparing the primary biological sequence information such as the nucleotides of DNA and/or ribosome-ribonucleic acid (RNA) sequences and amino-acid sequences of proteins. Thus, it was used to determine the similarity of the received assembled sequence of PCR product-insert (upon using the universal primer of pUC19) with the already submitted sequence in the data base. In addition, multiple sequence alignments were performed by using sequence retrieval system (SwissProt http://hcuge.ch/srs5/). SWISS-PROT is a curated protein sequence database which strives to provide a high level of annotation (such as protein-function description, domain structure, post translational modification … etc.), a minimal level of redundancy, and a high level of integration with other databases.

### Expression of recombinant cellulase gene by most active clone under lacz promoter

This was done by inoculating 50 ml of production LB broth medium (amp & IPTG) dispensed in 250 ml Erlenmeyer flask with 1 ml suspension from freshly prepared overnight pre-culture of the most active clone. A starting pre-culture was grown in 250 ml Erlenmeyer flask containing 50 ml of LB_amp_ medium under shaking (200 rpm) at 37 °C. After inoculation of the production medium, flasks were incubated at 37 °C under shaking (200 rpm); growth and cellulase activity were detected along 48 h. Growth was monitored by measuring the optical density (OD_600 nm_), while the cellulase activity was followed by measuring the color intensity (DNS assay) under the inducing conditions (IPTG, 1 mM). An additional flask was prepared and was run under the same conditions without IPTG to be used as control (non-induced) in the characterization of recombinant *cellSNSY* through sodium dodecyl sulfate–polyacrylamide gel electrophoresis (SDS-PAGE) and activity staining in the next experiment.

### Characterization of the recombinant protein cellSNSY

#### Protein estimation

In order to estimate the total soluble protein concentration, Lowry method was performed based on pre-prepared standard curve of bovine serum albumin [31].

#### SDS-PAGE and the activity staining (Zymography)

The recombinant protein samples (induced and non-induced at conc.10–20 μg) were treated with SDS-loading buffer, inactivated for 5 min at 95 °C, suspended on ice, and loaded onto the SDS-PAGE (12 %) according to Laemmli [32]. The gel run for 15 min at 20 mV in the electrode buffer, and then the voltage increased to 60 mV for approximately 3 h. After racing, the gel was stripped from the glass plates, divided into two sections; the first was subjected to stain by Coomassie then de-stained for protein visualization. The second section was incubated for 4 h at 4 °C in the renaturation solution (100 mM Tris–HCl buffer, pH 7.5 containing 0.5% Triton X-100) followed by 2 h at 50 °C in the substrate solution (50 mM Tris–HCl buffer, pH 7.5 containing 0.5% CMC) for cellulase activity detection. Next, the gel was placed in a Petri dish containing Congo-red solution (0.2% w/v) for 10 min at room temperature, and then, it was washed with 1 M sodium chloride. The existence of a faint yellowish halo band indicated that the CMC substrate had been hydrolyzed.

#### Determination of the optimum temperature

The optimum temperature of the crude recombinant *cellSNSY* was estimated over different temperatures (ranged 35–80 °C). The reaction was carried out under the initial assay conditions (pH 7.5 and shaking for 15 min).

#### Determination of the optimum pH

The optimum pH of the crude recombinant *cellSNSY* was considered upon testing a wide range of pHs (3–10). The experiment was designed by using citrate buffer (pH 3–5); phosphate buffer (6.0–7.0) and tris-HCl (pH 7.5–10) at 50 mM. The reaction assay mixtures were allowed to stand for 15 min under shaking at 45 °C incubation temperature.

#### Temperature stability

The thermal strength of the recombinant *cellSNSY* was measured at different temperatures ranging from 25 to 80 °C for (1–2 h), and then the temperature stability of the recombinant *cellSNSY* protein was checked at − 20 °C for up to 9 months without thawing. Furthermore, the stability was tested upon gradual weakly thawing of the tested recombinant protein which was kept in buffer (50 mM tris-HCl buffer pH 7.5) over 12 days at varied period breaks and the residual activity was determined. The reaction assay was carried out under the resulted optimal conditions and was expressed as % compared to untreated enzyme sample.

#### pH stability

The pH stability of the recombinant *cellSNSY* enzyme was checked at pH 7, 7.5, 8, and 8.5 for up to 9 months. The reaction assay was carried out under the resulted optimal conditions (45 °C, pH 8.5) and was expressed as % compared to untreated enzyme.

#### Effect of certain metals, solvents, surfactant, and EDTA on the enzyme activity

The crude recombinant protein *cellSNSY* was tested against different cations (MgCl_2_, CaCl_2_, ZnCl_2_, NaCl, FeSO_4_, MnSO_4_, and CuSO_4_), chelating agent (EDTA) at concentrations 1 mM, solvent (DMSO, methanol, ethanol, isopropanol, glycerol), and surfactant (SDS) at concentrations 1% (w/v). The tested enzyme was independently incubated with the tested agent for 15 min; the residual activity was calculated then subtracted from the activity of untreated sample to know the inhibition or activation % of the tested compound.

### Statistical analysis

All assays were performed in triplex reactions. The results were expressed as means ± standard deviation which was determined by using Microsoft Office Excel 2013.

## Results

### Metagenomic library construction

**Fig. 1:**
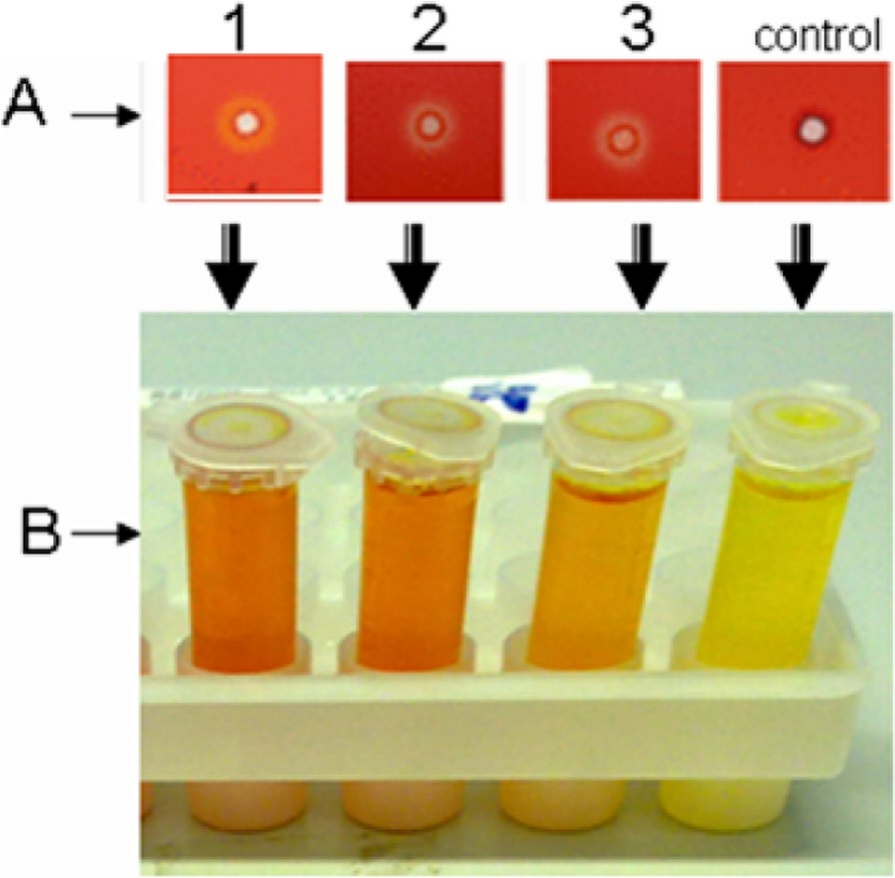
Activity screening for isolated recombinant cellulase clones (1, 2 and 3) derived from soil metagenomic. **A** Well cut method and **B** DNS method against negative control (buffer)

**Fig. 2:**
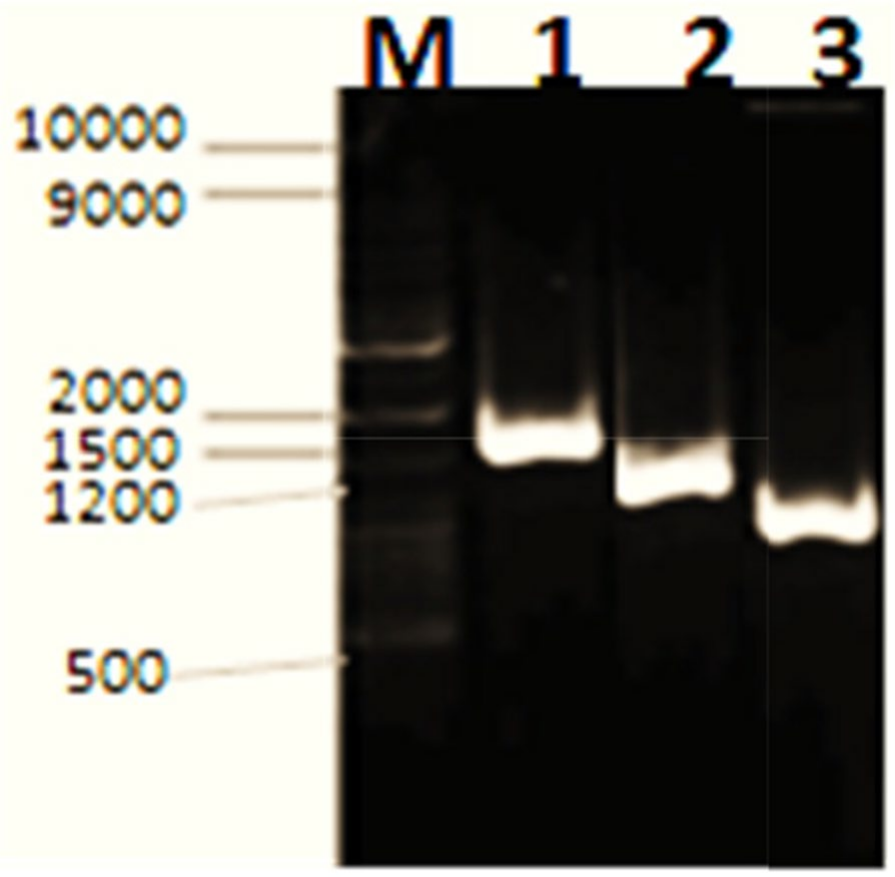
Agarose gel electrophoresis of the amplified PCR fragment- insert using M13 primer for different positive recombinant clones 1, 2, and 3. M: 10 kb DNA marker

**Fig. 3:**
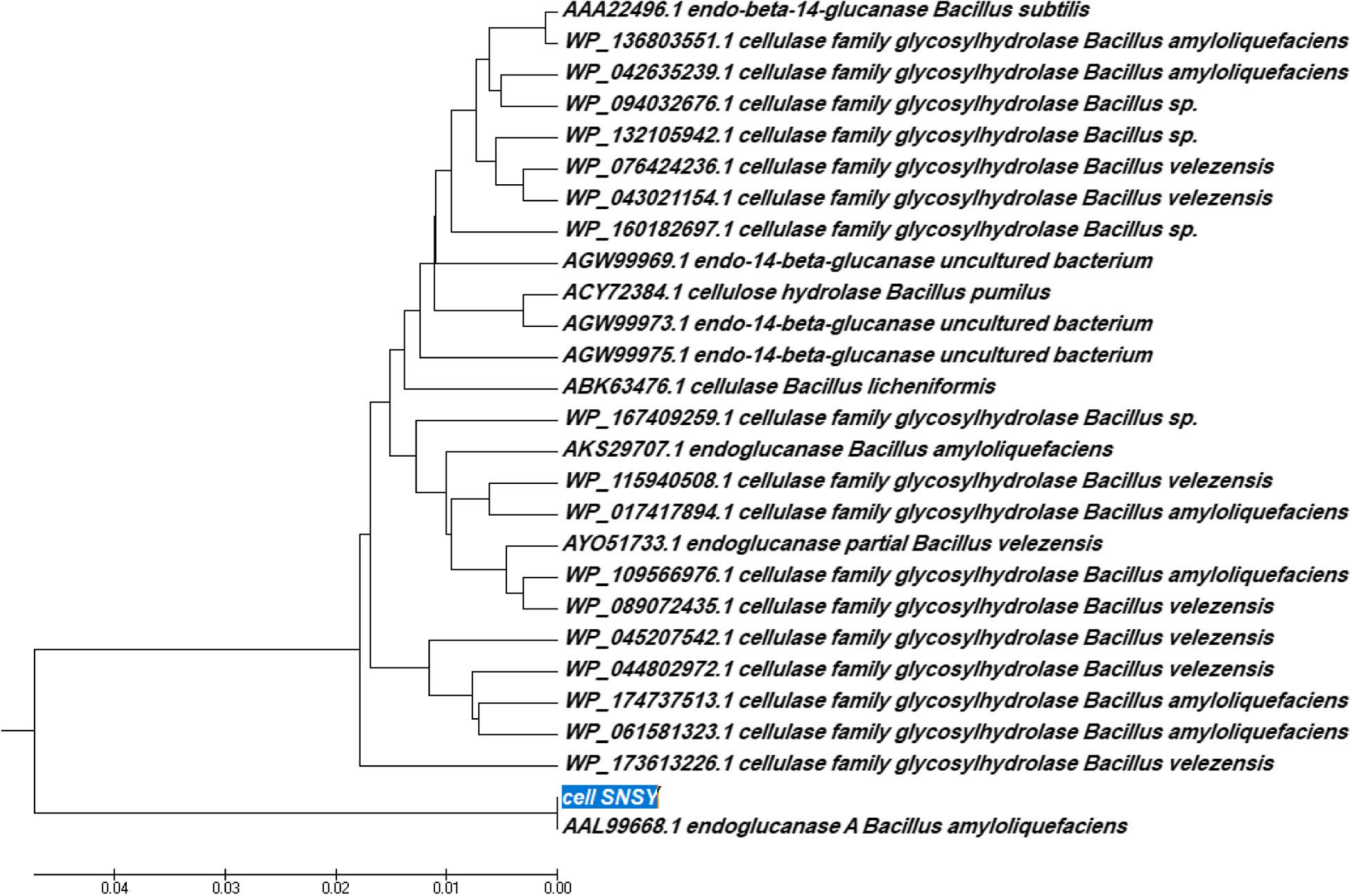
Phylogenetic relation of *cellSNSY* gene with cellulose(s) genes available in GenBank database. The dendogram was generated by the neighbor-joining method using MEGA7.0 Software

In order to clone positive cellulase clones from Wadi El Natrun, Egyptian soil, a metagenomic technique was applied. Initially, the work started with the isolation of total DNA from sediments collected from these extreme environmental conditions to use in library construction and transform onto *E*. *coli* DH5α. The extracted DNA reached to 14,361 ng/μl concentration with a high purity (1.82). Four independent soil-metagenomic libraries were constructed by using *Bam*HI, *Hind*III, *Eco*RI, and *Sal*I; 270 transformants clones in total were obtained from these libraries (78, 82, 99, and 11 clones), respectively. White transformant clones were picked and subcultured in a master plate; they were individually screened on LB_amp,IPTG-CMC_ agar plates that were incubated at 37 °C for CMC hydrolysis due to the expression of a heterologous cellulase. Three clones among all the tested clones had shown halo zone by plate assay (Fig. 1A). A quantitative estimation of the cellulase activity for these selected clones (1, 2, and 3) was carried out after 24-h cultivation in LB_amp, IPTG_-broth medium and was found to give such figures 19, 14, and 8 μ/ml , respectively (Fig. 1B). It is worth to mention that the transformant clone number 1 is derived from *Bam*HI-dependent library, while 2 and 3 from *Hind*III constructed library. These clones (1, 2, and 3) were picked from master plate and were cultivated in liquid LB_amp_ medium overnight for plasmid isolation. The related recombinant plasmids (pUC-clone 1, 2, and 3) contained an inserted DNA-fragment of about 1.5, 1.2, and 1 Kb as being determined by the PCR-reaction through using M13 flanking universal primer after running (agarose-gel electrophoresis) (Fig. 2). Based on these results, pUC-clone 1 insert was subjected to sequencing, where one consensus partial sequence of 861 bps was obtained after sequence assembly. These nucleotides were translated into amino acids by using BioEdit 7.2 software. The resulted partial sequence composed of 287 amino acids was compared to the data in National Center for Biotechnology Information (NCBI) GenBank via BLAST, where it showed 76% identity to cellulase family glucosyl hydrolase, namely endoglucanase gene of *Bacillus amyloliquefaciens* (GenBank accession no. AAL99668). The phylogeny of the metagenomic isolated gene designated *cellSNSY* (287aa) and the closely related genes was analyzed by using the multiple-sequence alignment program (CLUSTALW, Pairwise Alignment), and the results were presented in a phylogenetic tree as shown in Fig. 3.

### Heterologous expression of cellSNSY under lacz promoter

**Fig. 4:**
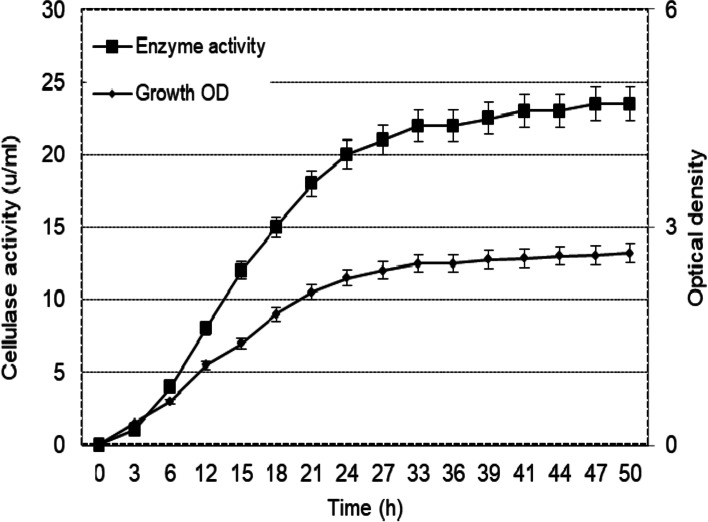
Growth course versus enzyme activity upon heterologous expression of cellSNSY under *lac*z promoter cultured in LB_amp IPTG_. broth medium along 2 days incubation under sacking at 37 °C

After the cloning of *cellSNSY* gene (namely endoglucanase), its expression was monitored under *lac*z-promoter using IPTG (1 mM) (Fig. 4). It was noticed that the growth and the cellulase activity of the recombinant *cellSNSY* were progressively increasing by time elongation up to 27 h (the end of exponential growth phase) with no noticeable increase either in growth or activity.

### Characterization of the recombinant cellSNSY

**Fig. 5:**
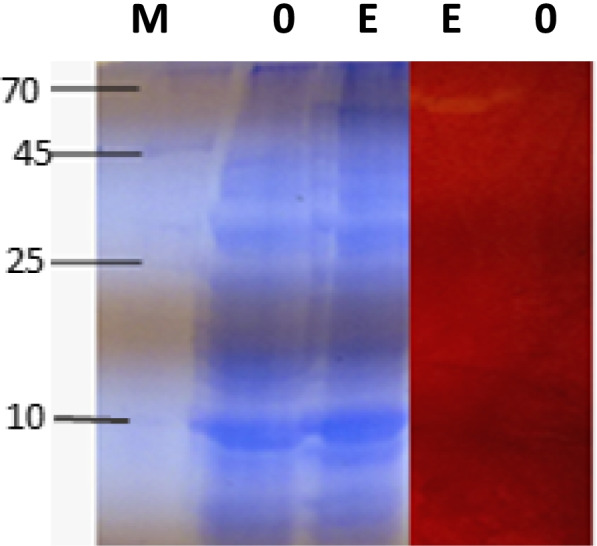
SDS-PAGE (12%) and activity staining (zymogram) for cellSNSY recombinant clone 1 (cell lysate). Lane M: protein marker, left side (stained with Coomassie stain) and right side (zymogram). Lane 0, E: non-induced and IPTG induced recombinant cellSNSY samples, after 24-h incubation in LB_amp_ broth, respectively

Quick analysis of recombinant proteins derived from *cellSNSY* (cultivated under induced and non-induced conditions) was performed through SDS-PAGE and activity stain. It was recognized in Fig. 5 the appearance of an active band at molecular weight ~ 59 kD by inducing sample.

### Effect of temperature

**Fig. 6:**
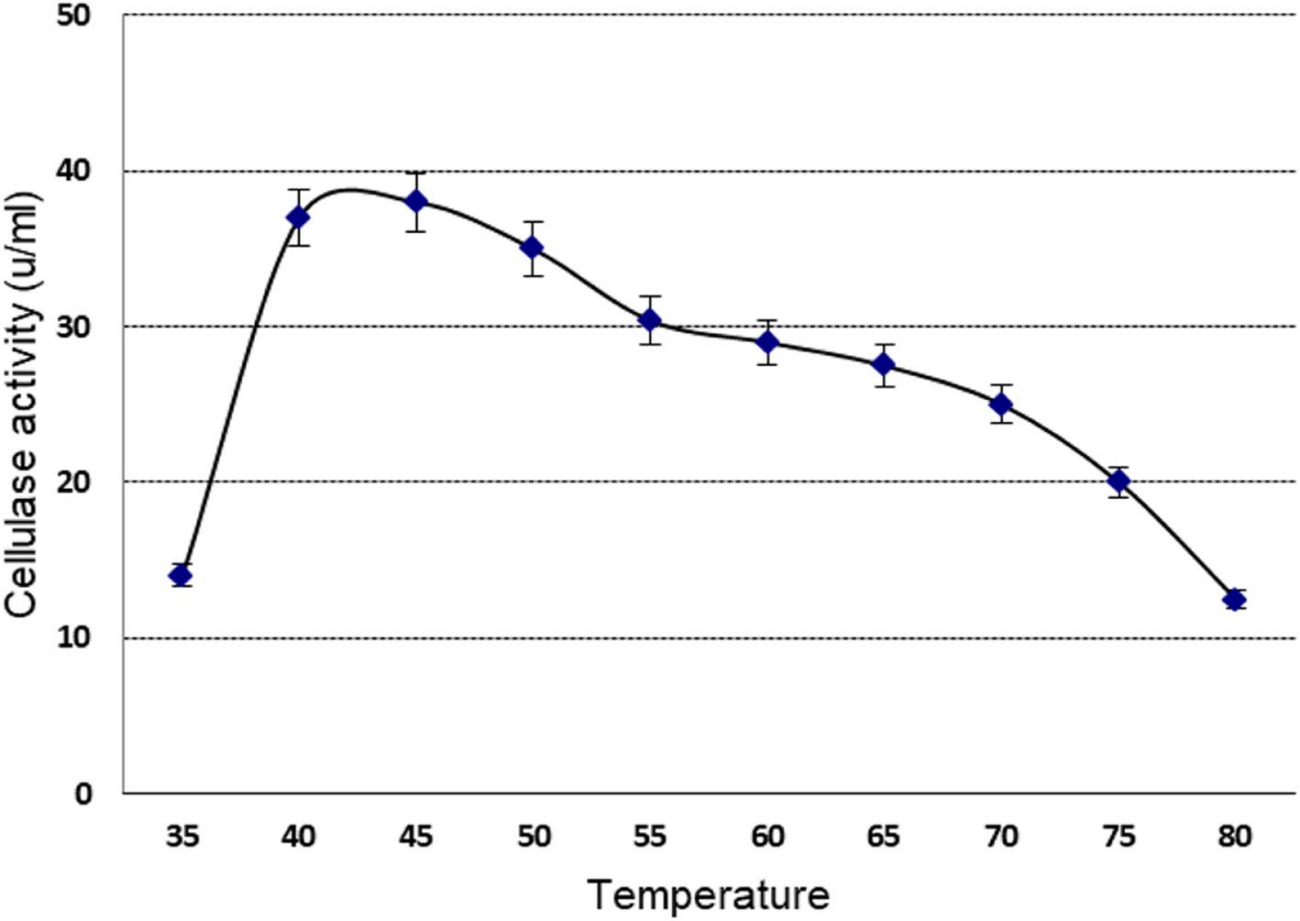
Optimum temperature of the recombinant cellSNSY enzyme

**Fig. 7:**
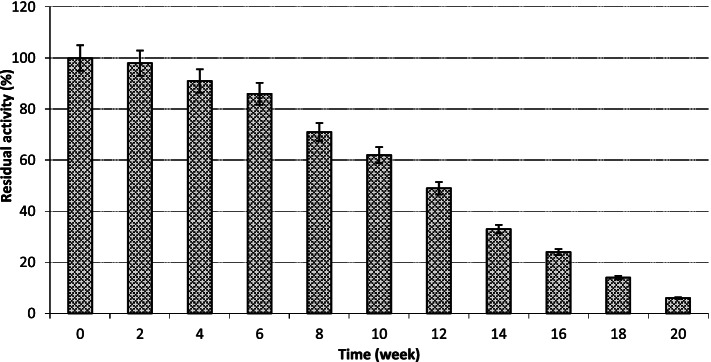
Effect of thawing on recombinant cellSNSY enzyme activity

As described in the “Methods” sections, different temperatures (35–85 °C) were tested to determine the optimal value for the recombinant *cellSNSY*. The data plotted in Fig. 6 explained that the optimal activity had appeared at 45 °C. On the other hand, the recombinant *cellSNSY* showed a complete stability at temperatures (20, 30, 35 °C); (40, 45 °C); and (50, 55 °C) for 4 h, 2 h, and 50 min (data not shown), respectively. Whereas enzyme exposure to higher temperatures (55, 60, and 70 °C) caused noticeable drop in the thermal stability of the recombinant *cellSNSY* which were approximately equal 14, 34, 42, and 49% (data not shown) after 15-min exposure, respectively. This means the half-life of crude recombinant *cellSNSY* was 15 min at 70 °C. On the other hand, stability of the recombinant *cellSNSY* was tested upon being freezed at − 20 °C (without thawing condition) throughout 20 weeks with 2 weeks intervals (Fig. 7). Data in this figure indicated that the recombinant *cellSNSY* was highly stable under such freezing condition and 30% of enzyme activity was lost after 8 weeks. However, time extension caused continuous drop in the retained activity and 50% loss had resulted after 12 weeks as shown in Fig. 7. In addition, the stability was checked upon gradual weakly thawing (up to 10 times freezing/thawing) of the recombinant protein (*cellSNSY*) samples along 20 days where 94% loss in the enzyme activity of *cellSNSY* was noticed after ten times (data not shown).

### Effect of pH

**Fig. 8:**
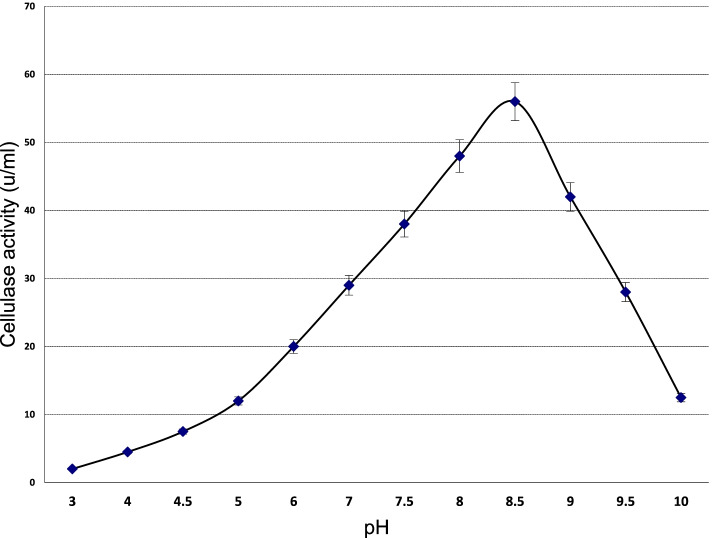
Optimum pH of the recombinant cellSNSY enzyme

The crude recombinant *cellSNSY* enzyme activity rate was studied as a function of the pH ranging from 3 to 10. The results presented in Fig. 8 explained that the recombinant *cellSNSY* had worked in an alkaline condition giving its optimal at pH 8.5.

**Fig. 9:**
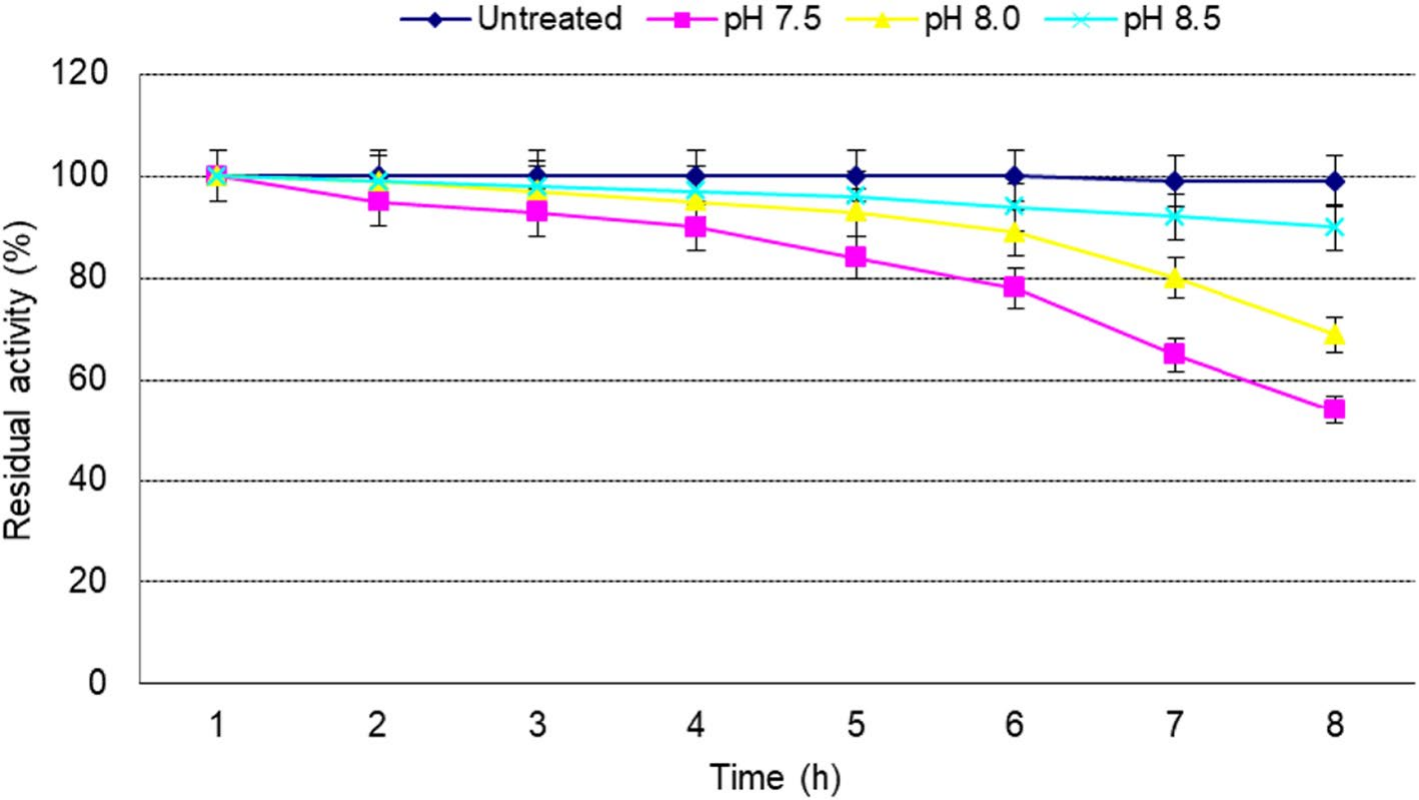
Monitoring of pH stability for recombinant cellSNSY enzyme activity through long-term preservation at − 20 °C without thawing

The pH stability of the recombinant *cellSNSY* was monitored in an alkaline condition (pH 7.5, 8.0, and 9.0) along 240 min (4 h) at room temperature (data not shown) where it showed complete pH stability at these tested pH. Alongside, the *cellSNSY* recombinant stability was tested upon the preservation at different pHs (7.5, 8.0, and 8.5) and under cooling (− 20 °C) for 8 weeks and was compared to untreated enzyme. The data illustrated in Fig. 9 indicated that the tested *cellSNSY* withstood the preservation under alkaline pH 8.5 > 8.0 > 7.5. The highest loss in activity % (50, 35, and 15) occurred after 8 h of preservation at pH 7.5, 8.0, and 8.5, respectively.

### Effect of different compounds

Metal ions can be binder to proteins and to other enzyme-related molecules to form complexes. The effect of different cations, surfactants, solvents, and metal chelators were tested and the response of *cellSNSY* toward the tested substances was reported and was shown in Table 1. The outcomes indicated that the presence of MgCl2, CaCl2, NaCl, glycerol, DMSO did not decrease the activity of *cellSNSY* at all. ZnCl2, ethanol, and isopropanol showed very limited decrease in activity (ranged 1–2%). Moreover, a noticeable decrease in % of the residual activity around (30, 15, 13, and 13) was caused by treating the investigated enzyme with the following heavy metals: CuSO_4_, MnSO_4_, FeSO_4_, and surfactant (SDS), respectively as shown in Table 1.

**Table 1:** Effect of some metal ions, SDS, solvents, and inhibitors on the activity of recombinant cellSNSY enzyme activity

Residual activity of recombinant cellSNSY activity (%)Mean ± SD	
**Tested**	**Different metal ions**
**Cu**^**+2**^	71.32 ± 0.5
5.1.1.1. **Ca**^**+**^	101.42 ± 1.2
5.1.1.2. **Na**^**+**^	100.54 ± 1.3
5.1.1.3. **Mg**^**+2**^	102.1 ± 0.99
5.1.1.4. **Mn**^**+2**^	85.. ± 0.8
5.1.1.5. **Fe**^**+2**^	87.5 ± 1.34
5.1.1.6. **Zn**^**+2**^	99.55 ± 2.2
5.1.1.7. **Control (untreated enzyme)**	100 ± 0.0
	**Surfactants and solvents**
5.1.1.8. **SDS**	87.7 ± 0.8
5.1.1.9. **Methanol**	94.1 ± 1.2
5.1.1.10. **Ethanol**	99.7 ± 0.82
5.1.1.11. **Glycerol**	100 ± 1.0
5.1.1.12. **Isopropanol**	98.2 ± 1.4
5.1.1.13. **DMSO**	100.6 ± 0.8
5.1.1.14. **Control (untreated enzyme)**	100 ± 0.0
	**Metal chelate**
5.1.1.15. **EDTA**	95.5.5 ± 2.2
5.1.1.16. **Control (untreated enzyme)**	100 ± 0.0

## Discussion

Cellulose is considered one of the most abundant agriculture wastes. It constitutes the major structural components of the cell walls of higher and lower plants. Huge amounts are produced annually, but its accumulation is problematic. This study had been directed to look for a new cellulase enzyme with specific properties to be useful in many applicable fields. In order to reach this target, the study aimed for cloning a positive cellulase recombinant (s) from Wadi El Natrun, Egyptian, soil by implementing a metagenomic technique. Initially, the work started with the isolation of total DNA from soils which were collected from the extremely saline and alkaline lakes of the Wadi El Natrun to be used in library construction and to be transformed onto *E*. *coli* DH5α. Soda lakes are found all over the world and are classified as a highly productive environment, though, being extreme. Such locales are considered good supplies for novel species of microbes; the extremophilic nature of these microorganisms will make them a potential source for enzymes and metabolites with industrial uses [33]. Most of the microbes survived in extreme environment and are difficult to be cultured in the laboratory. Therefore, metagenomic technique (culture-independent method) facilitates the isolation of novel enzymes (genes) from the total microbial population including the unculturable one by using material extracted directly from environmental samples [34]. Recently, a wide variety of hydrolases have been cloned from environmental DNA via applying metagenomic technique [35–38].

A successful metagenomic creation mainly depends on DNA quality and concentration where the low DNA concentration, difficulty in extraction, and cloning are major obstacles. In this study, soil DNA collected from saline and alkaline lakes of the Wadi El Natrun was successfully extracted (conc. 14,361 ng/μl) with a good purity (1.82). Afterwards, soil metagenomic library was constructed by using the restriction enzymes (*Bam*HI, *Hind*III, *Eco*RI, and *Sal*I) and 270 transformants clones that were obtained as based on blue/white screening in the presence of X-gal and IPTG. The obtained numbers of transformants (270 clones) indicated a low cloning efficiency result which needed to be improved. This might be related to the presence of some inhibitors like humic acid in environmental samples. Humic acid was found as a coextracted; its existence caused an inhibition of nucleases restriction enzyme and a limitation in transformation processes [39].

Cellulolytic activities of liberated clones were monitored; three active clones were recognized by a yellow halo zone of hydrolysis (plate assay). The most active clone (clone 1) with the maximum cellulase activity was further identified as a novel cellulase gene via analysis of the nucleotide and amino acid sequence. A partial amino acid sequence of *cellSNSY* (287aa) showed 76% identity to cellulase family glucosylhydrolase, namely endoglucanase gene of *Bacillus amyloliquefaciens* (GenBank accession no. AAL99668). In general, cellulase is not a single enzyme (a group composed of endoglucanase and exoglucanases) that are including cellobiohydrolases and β-glucosidase. These classes work in synergistic way in order to completely hydrolyze the cellulose molecule [38]. As far as it is concerned here, this is the first report about the isolation of endoglucanase related cellulase gene through metagenomic library from Wadi El Natrun by functional screening. The *cellSNSY* gene derived from metagenomic library was cloned and was expressed under *lac*z promoter in *E*. *coli* DH5 using IPTG (1 mM) where its crude protein was characterized. The expressed *cellSNSY* showed an active band with a molecular mass (~ 59 kDa) through zymography (in situ detection of cellulolytic activity). This obtained molecular weight was nearer the cellulase from a thermophilic *Paenibacillus barcinonensis* 58.6 kDa [40], and that from *Bacillus mycoides* (62 kDa) [41]. Consequently, the crude recombinant *cellSNSY* was subjected to intensive characterization to explore its uniqueness in traits which further nominated it for some specific applications. The maximum activity of *cellSNSY* was recorded at 45 °C. This result agreed with the results obtained by [42] who found the optimum temperature for *Aspergillus niger* MK543209 cellulase as being 45 °C. Li et al. [43] reported that most thermophiles were having optimum temperature ranging between 65 and 70 °C. Therefore, it was recognized that about 45% of cellulose degradation have occurred due to the action of bacterial consortia at 37 °C [44]. Patel et al. reported the novel cellulase named *Cel-5M* from rumen metagenome which showed maximum activity at 40 °C [45]. Thermal stability of enzymes is considered a very important feature for industrial applications. By testing *cellSNSY*, it was found to be completely stable at tested temperatures 25–55 °C for about 1 h. However, it lost ~ 50% of its activity at 70 °C after 15 min of exposure. Patel et al. [45] recorded the novel recombinant *Cel*-*5M* retained 65% for its activity by measuring the thermal stability between 30 and 70 °C. Ogonda et al. [46] demonstrated that the cellulase enzyme of *Bacillus* sp. had retained approximately 99 and 40% of activity at 60 °C and 80 °C, respectively. Islam et al. [47] stated that about 68% of the activity had been retained after heating the crude cellulase enzyme solution from *Bacillus* sp. at 50 °C for 30 min. These results were in contrast with those reported by Li et al. [48] who stated that cellulose produced by *Bacillus tequilensis* strain GYLH001 from Angelica can remain active even it was heated at 100 °C for 30 min. It is worth mentioning that cellulases are characterized by a high thermal stability that designates them for rendering for sustainable agriculture, cellulose-based research, and industrial processes.

Likewise, *cellSNSY*, temperature stability was monitored also under freezing and the enzyme showed a complete stability up to 8 weeks. This means that the enzyme is able to survive for long term preservation under freezing conditions.

As it was previously reported, the *cellSNSY* under investigation had indicated preferable tolerance to act in an alkaline condition where the optimum was recorded at pH 8 by showing complete pH stability under alkaline conditions (pH 7.5–8.5). This finding is in contrast with the recombinants designated *Cel*-*5M* and *Cel14b22* which showed maximum activities at pH 6.0 and 6.0–7.0 [45, 49], respectively. Also, optimum pH was found to be 6.5–7.5 for the cellulose-degrading bacteria which were isolated from Saliva Breed of Buffalo [50]. To add more, a metagenomic-derived *Cel5M* had retained more than 80% activity between 4 and 7 pH as described by Patel et al. [45]. Some researchers stated that cellulases are generally stable over a wide range of pH from 5.0 to 10.0 [51, 52], especially thermophiles are usually having an optimum pH that ranged (pH 6–10) [43]. Gong et al. [49] found that the recombinant metagenomic cellulases have a broad stability that are ranging from pH 4.0 to 10.0.

It is worth to mention that the increasing or the decreasing the pH changes the ionic state of ionizing side chains of protein molecule, disrupts ion pairs, breaks hydrogen bond, and consequently denatures the protein [53]. In contrast to results given here in this study, the previous findings reported that acid cellulase from *Aspergillus niger* showed the highest activity at pH 2.5 [54]. Maleki et al. [55] have been trying to develop a cocktail (*PersiCell 1&2*) of novel thermostable cellulases with high hydrolytic ability and stability as the first *PersiCel1* works optimally at pH 8.0 and the *PersiCel2* optimally active at the pH of 5.

Metal ions can form complexes in association with proteins and other molecules that are related to enzymes. They may act as donors or acceptors of the electron as structural regulators [56].

The effect of different cations, solvents, surfactant, and EDTA on the crude recombinant enzyme (*cellSNSY*) activity was tested. The results showed that the presence of cations such as Mg^2+^, Ca^2+^, Na^+^, and Zn2^+^ did not decrease the activity. Fe^2+^, Mn^2^, and Cu^2+^ caused a decrease in the *cellSNSY* enzyme activity. These results agreed with the results obtained by many researchers [57–59], who reported that both Ca^2+^ and Na^+^ cations exhibited stimulatory effects on the enzyme activity in all tested concentrations. The results of the under investigation are in agreement with Asha et al. [40] who reported for the activity of the purified cellulase from *Paenibacillus barcinonensis* which is stimulated in the presence of metal ions such as Mg^2+^, Mn^2+^, and Co^2+^. Also, Chai et al. [22] found that the cellulase activity was increased by Mn^2+^, Ca^2+^, Zn^2+^, and Mg^2+^ at 1 mM. In contrast to ones’ results, Akintola et al. [57] reported that Mg^+2^ inhibited the activity of cellulase from *Enterobacter cloacae* IP8 at concentration 4 mM to 15 mM; it stimulated its activity at concentrations (ranged 20–200 mM). Gong et al. [49] noticed that the recombinant *Cel14b22* was considerably enhanced by Mn^2+^, but intensely reduced by Fe^3+^ or Cu^2+^

By testing EDTA (1 mM), the studied recombinant *cellSNSY* lost 5% of its activity, while Akintola et al. [57] reported that EDTA at concentrations above 4 mM inhibited the activity of the crude cellulase from the bacterium (*Enterobacter cloacae* IP8). Similarly, EDTA caused the inhibition of cellulase which was produced by *Acetobacter xylinum* Ku-1 [60]. EDTA as a metal chelating agent probably was acted by the inactivating of the cellulase either by removing metal ions from the enzyme through the formation of coordinating complex, or by binding them inside the enzyme as a ligand as had been noted by Schmid [61].

Additionally the recombinant *cellSNSY* showed a resistance to the tested solvents like DMSO, alcohols, and glycerol, although the surfactants (SDS) had been caused around 15% drop in the activity. By the same token, SDS surfactant caused around 34% of inhibition in cellulase activity from *Acinetobacter junii* GAC 16.2 [62].

## Conclusions

Wadi El Natrun microorganisms have been considered a pronounced source for discovery of new natural products and metabolites. Thus, a functional metagenomic route was followed for cloning a novel cellulose gene from environmental soil samples collected from Wadi El Natrun. Samples existed under extreme. Saline and alkaline were selected to prospectively find an enzyme that can be able to withstand the harsh conditions. A successful soil-metagemnomic library was developed, but low transformation efficiency was recognized. Activity screening for transformants had discriminated many active clones regarding cellulose hydrolysis by plate assay and the monitoring of the liberated reducing sugars (DNS colorimetric assay method). Moreover, the most active clone designated *cellSNSY* had shown an active protein band at molecular mass ~ 59 kD through SDS-PAGE and activity staining. Molecular characterization for *cellSNSY* had shown 76% identity to cellulase family glucosylhydrolase, namely endoglucanase. Characterization of crude recombinant protein explained that the *cellSNSY* works optimally at 45 °C and pH 8.5. Its half-life is 15 min at 70 °C. It showed great stability under alkaline conditions, and it is suitable for long-term preservation under freezing conditions. It showed complete stability toward the tested alcohols, DMSO, glycerol, and heavy metals like Zn^+2^. The highest inactivation (~ 30%) caused by Cu^2+^ followed by Mn^2+^ (~ 15%) and Fe^+2^ (~ 13%). A 1% surfactant (SDS) postponed the activity by 12%. The overall features and properties of the studied *cellSNSY* made it suitable for many future applications in industries; like textile, paper, and cellulose dependent researches.

## Data Availability

All data generated or analysed during this study are included in this published article.

## Abbreviations

CMC: Carboxymethylcellulose
DMSO: Dimethyl sulfoxide
EDTA: Ethylene diamine tetra acetic acid
SDS-PAGE: Sodium dodecyl sulfate–polyacrylamide gel electrophoresis
SDS: Sodium dodecyl sulfate
amp: Ampicillin
X-gal: 5-bromo-4-chloro-3-indolyl-β-D-galactopyranoside
IPTG: Isopropyl β-d-1-thiogalactopyranoside
DNS: Dinitrosalicylic acid
PEG: Polyethylene glycol
RNA: Ribosome-ribonucleic acid
DNA: Deoxyribonucleic acid
PUC: Plasmid cloning vector
PCR: Polymerase chain reaction
BLAST: Basic Local Alignment Tool
NCBI: National Center for Biotechnology Information
MEGA: Molecular evolutionary genetics analysis
aa: Amino acids
OD: Optical density
TSS: Transformation storage solution
*E. coli*: *Escherichia coli*
U: Units
w/v: Weight/volume
h: Hour(s)
min: Minute(s)
sec: Second(s)
rpm: Rotation per minute
bp: Base pair
kDa: Kilo Dalton
Kb: Kilobase pair
LB: Luria-Bertani
NA: Nutrient agar
